# Benzoyl chloride derivatization improves selectivity and sensitivity of lipidomic quantitation in human serum of pancreatic cancer patients using RP-UHPLC/MS/MS

**DOI:** 10.1007/s00216-025-06151-0

**Published:** 2025-10-06

**Authors:** Ondřej Peterka, Zuzana Lásko, Robert Jirásko, Petra Peroutková, Anna Taylor, Beatrice Mohelníková-Duchoňová, Irena Kozubíková, Martin Loveček, Bohuslav Melichar, Michal Holčapek

**Affiliations:** 1 Department of Analytical Chemistry, Faculty of Chemical Technology, University of Pardubice, Studentská 573, 532 10 Pardubice, Czech Republic; 2 Department of Oncology, Faculty of Medicine and Dentistry, Palacký University Olomouc and University Hospital, Zdravotníků 248, 775 20 Olomouc, Czech Republic; 3 Department of Surgery, Faculty of Medicine and Dentistry, Palacký University Olomouc and University Hospital, Hněvotínská 3, 779 00 Olomouc, Czech Republic

**Keywords:** Derivatization, Lipidomic quantitation, Liquid chromatography, Mass spectrometry, Human serum, Pancreatic cancer

## Abstract

Chemical derivatization is a powerful strategy for enhancing the chromatographic behavior and mass spectrometric sensitivity of lipids, which play an essential role in cellular processes and show high potential in cancer biomarker research. In this study, we describe a targeted and validated method that combines benzoyl chloride derivatization with reversed-phase ultrahigh-performance liquid chromatography tandem mass spectrometry (RP-UHPLC/MS/MS) for the quantitative analysis of the human serum lipidome. In total, 450 lipid species from 19 lipid subclasses were identified based on a combination of multiple reaction monitoring transitions, retention dependencies, dilution series, and derivatization tags. The developed methodology increases the sensitivity for most investigated lipid classes in comparison to conventional methods, but the highest improvement was observed for monoacylglycerols, diacylglycerols, sphingoid bases, and free sterols. The method’s accuracy was confirmed using NIST SRM 1950, as the determined concentrations were in agreement with the consensus values from ring trials. Lipidomic profiling of clinical samples revealed a significant dysregulation of lipid metabolism in pancreatic cancer patients compared to healthy controls. Key findings included the upregulation of most monoacylglycerols and sphingosine, and a pronounced downregulation of sphingolipids with very long saturated *N*-acyl chains and phospholipids containing fatty acyl compositions 18:2 and 20:4. This targeted approach is consistent with the trends previously seen with other methods and also provides new findings and more detailed structural insights into metabolic alterations in pancreatic cancer.

**Supplementary Information:**

The online version contains supplementary material available at 10.1007/s00216-025-06151-0.

## Introduction

Lipids are highly diverse biomolecules, and the LIPID MAPS database contains more than 49,000 lipid structures divided into categories, classes, and sub-classes [1]. Lipids play an indispensable role in cellular homeostasis, and alterations in lipid profiles have been associated with various serious disorders, such as cardiovascular diseases [2], neurodegenerative diseases [3], and cancers [4]. Lipidomics is a branch of omics science that studies the structure, function, and metabolism of lipids. Pancreatic ductal adenocarcinoma (PDAC) is one of the most aggressive and deadliest malignancies, with a 5-year relative survival rate of only 13% [5] and it is predicted that PDAC will become the second leading cause of cancer-related deaths by 2030 [6]. The identification of biomarkers that would facilitate the early diagnosis or provide information leading to the discovery of new therapeutic targets remains an unmet medical need. Lipid biomarkers for distinguishing healthy controls and PDAC patients were proposed using methods based on the lipid class separation [7, 8], but information based on the fatty acyl level is necessary for the biological interpretation of lipidomic data and understanding of lipid metabolism.

Ultrahigh-performance liquid chromatography-mass spectrometry (UPHLC/MS) is the most widely used approach in lipidomic analysis. The combination of both techniques allows for the separation of isomeric compounds, structural analysis, and high-throughput quantitation. Although several LC/MS platforms have been successfully used in targeted lipidomic analysis, LC with triple quadrupole instruments is still the most popular due to the high sensitivity provided by multiple reaction monitoring (MRM) [9, 10]. Class-selective fragments are common for lipid species belonging to the same lipid class, such as *m*/*z* 184 for phosphatidylcholines (PC) and sphingomyelins (SM) or the neutral loss of Δ*m*/*z* 141 for phosphatidylethanolamines (PE). However, some lipid classes do not provide selective MRM transitions, such as free sterols (ST), sphingoid bases (SPB), fatty acids (FA), and monoacylglycerols (MG), leading to lower detection sensitivity using single ion monitoring or non-selective transitions, such as the neutral loss of water [11]. Moreover, neutral losses and precursor ions of fatty acids for quantitative analysis can lead to inaccurate concentrations due to large differences in responses of individual FA and the presence of two or more FA with the same composition [12, 13]. These limitations are observed especially for neutral lipids and can be compensated by response factors [10, 14].

Chemical derivatization is mainly used for improving retention behavior and sensitivity, but it can also bring additional parameters for the high confidential identification and the quantitative analysis using derivatization tags. Lipids mainly include carboxyl, hydroxyl, and amino functional groups. Therefore, many reaction mechanisms can be used for the derivatization [15]. The derivatization is mostly focused on the selective reaction for individual lipid classes, such as fatty acids [16], glycerolipids [17], phospholipids [18], sphingolipids [19], and free sterols [20, 21], but highly reactive derivatization agents can be applied for multiple lipid classes [22, 23]. In addition, there is an approach to using isotopically labeled derivatization reagents for the relative quantitative analysis to obtain an internal standard (IS) for each derivatized analyte after mixing unlabeled and labeled derivatized samples [24]. The localization of double bond positions due to the subsequent formation of diagnostic fragment ions is an important application of derivatization reactions [25].

The aim of this study is the development of a quantitative lipidomic method combining the chemical derivatization and lipid species separation approach. The methodology is mainly focused on lipid species without characteristic MRM transitions by conventional approaches, while maintaining the quality of quantitation for other lipid species. The optimized and validated method is used for the quantitative analysis of NIST Standard Reference Material 1950 (NIST SRM 1950) human plasma and compared with the literature values. Lipidomic profiling of PDAC patients and healthy controls is investigated to illuminate the metabolic behavior of often neglected lipid classes, such as MG, diacylglycerols (DG), SPB, and ST, which lie at the metabolic crossroads and can play an important role in the cancer mechanism.

## Materials and methods

### Chemicals and standards

Benzoyl chloride (Bz-Cl) 99%, pyridine (for HPLC, ≥ 99.9%), ammonium formate (for MS, ≥ 99.0%), and LiChrosolv chloroform (stabilized with 2-methyl-2-butene) were purchased from Merck (Darmstadt, Germany). Methanol (MeOH), acetonitrile (ACN), 2-propanol (IPA), formic acid (all LC/MS gradient grade), and ammonium carbonate (≥ 30.0% NH_3_ basis) were bought from Honeywell (Charlotte, NC, USA). Deionization water was prepared by the Milli-Q Reference Water Purification System (Molsheim, France). IS (Table S1) were obtained from Avanti Polar Lipids (Alabaster, AL, USA), Nu-Chek Prep (Elysian, MN, USA), or Merck (Darmstadt, Germany). All stock solutions of lipid standards were prepared in MeOH/CHCl_3_ (1:1, *v/v*). Deuterated IS are typically delivered in chloroform solution, which were directly used as a stock solution. All stock solutions were stored at − 80 °C.

### Human samples

For the method optimization, identification, and validation, pooled serum sample was prepared by mixing 60 human serum samples from healthy volunteers without a cancer diagnosis (ages 40–70 years and body mass index (BMI) of 20–30). Samples of 30 males and 30 females (Table S2) were obtained from Palacký University and University Hospital Olomouc, Czech Republic. The NIST SRM 1950 human plasma was used as a reference material for the quantitative analysis and the correlation with literature concentrations. For the lipidomic profiling of healthy controls (N) and PDAC patients (T), serum samples from 22 N and 22 T males (aged 40–75 years and BMI of 20–36) were collected at Palacký University and University Hospital Olomouc (Czech Republic). The clinical information for each subject is summarized in Table S3. All samples were stored at −80 °C until the lipidomic extraction. All subjects in the study were over 18 years of age, and blood samples were collected from each volunteer after overnight fasting. This study was carried out in accordance with the Declaration of Helsinki. Informed consent was obtained from all volunteers, and the ethical committee approved the collection of blood samples.

### Sample preparation

Protein precipitation of 10 μL human serum spiked with 20 μL of internal standard mixture (IS-Mix) was performed using 250 μL of CHCl_3_/MeOH/H_2_O mixture (30:60:8, v/v/v) [26] and placed in an ultrasonic bath for 10 min at 30 °C. After cooling to room temperature, 500 μL of CHCl_3_/MeOH/H_2_O mixture was added, and the samples were centrifuged (Hettich EBA 20) at 6000 rpm (3 462 × g) for 5 min. The supernatant was collected into a 4-mL glass vial and evaporated using a gentle nitrogen stream at 35 °C. The residues were stored at −80 °C for future experiments or directly used for the derivatization.

The optimized reaction using benzoyl chloride was applied for the derivatization of the lipids [22]. The sample residue was redissolved in 335 µL of pyridine (1:9 Pyr/ACN, v/v), and then 120 µL of benzoyl chloride (1:9 Bz-Cl/ACN, v/v) was added. The reaction mixture was slowly stirred at 320 rpm (KS 130 shaker, IKA, Staufen, Germany) at ambient temperature for 60 min. Afterwards, the reaction was terminated, and the excess of derivatization reagents was removed by employing a modified Folch lipid extraction protocol [27]. To the reaction mixture, 3 mL of CHCl_3_/MeOH (2:1, v/v) and 0.6 mL of 250 mM ammonium carbonate were added and stirred at 560 rpm (KS 130 shaker) for 5 min at room temperature. Then, the samples were centrifuged at 6000 rpm for 3 min, and the organic layer was collected and evaporated under a gentle stream of nitrogen at 35 °C. Just before LC/MS analysis, the residues were dissolved in 250 μL of CHCl_3_/MeOH (1:1, v/v).

### RP-UHPLC/MS/MS conditions

The RP-UHPLC/MS/MS method was used for lipidomic analysis using the lipid species separation approach. The Agilent 1290 Infinity series liquid chromatograph (Agilent Technologies, Waldbronn, Germany) was connected to a low-resolution hybrid quadrupole-linear ion trap (QTRAP) 6500 mass spectrometer (SCIEX, Framingham, MA, USA). The RP-UHPLC method [22, 28, 29] used the following conditions: Acquity UPLC BEH C18 column (150 mm × 2.1 mm, 1.7 μm), flow rate 0.35 mL/min, column temperature 55 °C, injection volume 2.5 μL, and autosampler temperature 4 °C. The linear gradient elution was used: 0 min – 35% B; 8 min – 50% B; 21 min – 95% B; 23 min – 95% B; 24 min – 35% B; 25 min – 35% B with 2 min post-run before the next injection. The mobile phase A was a mixture of acetonitrile-water (60/40, v/v) and the mobile phase B was a mixture of acetonitrile-2-propanol (10/90, v/v), with both phases containing 0.1% formic acid and 5 mM ammonium formate. The needle wash program started 23 min after each injection and involved both an external wash through the flash port and an internal wash by drawing and ejecting the maximum volume of IPA/MeOH/CHCl_3_ (4:2:1, v/v/v) with 5% H_2_O. During the needle wash program, the injector was set to bypass mode.

The mass spectrometer was equipped with a Turbo V ion source, and measurements were carried out in positive ion mode. The instrument was operated using the following optimized settings: capillary voltage 5.5 kV, drying temperature 250 °C, curtain gas pressure 20 psi, nebulizer gas pressure 50 psi, heating gas pressure 50 psi, and acquisition *m*/*z* range 50–1200. The collision energy and the declustering potential (DP) were optimized for individual lipid classes using IS. All MS parameters are summarized in Tables S4. To eliminate contamination of the ionization source by the residual derivatization agents, a divert valve was used to bypass the ion source of the mass spectrometer, directing it to waste during the interval from 0.1 to 1.8 min.

### Data processing

All data were acquired using Analyst software (version 1.6.2) from SCIEX, and Skyline software [30] was employed to determine the peak areas of individual lipids. The identification was performed manually using an in-house database of lipids. Precursor ion scans (PIS), neutral loss scans (NLS), and MRM scans based on characteristic fragment ions, with confirmation by retention dependencies, were used. The method validation was evaluated based on peak areas of IS and calibration curves; limit of quantitation (LOQ), limit of detection (LOD), repeatability, accuracy, precision, selectivity, carry-over, and instrument precision were investigated. Lipid concentrations were determined using IS added to the sample prior to extraction. The concentration of each lipid species was calculated by dividing its peak area by the peak area of the corresponding IS and then multiplying the result by the known concentration of the IS. Lipid species with determined concentrations for at least 75% of all samples were included in the dataset, and zero filling was applied for missing values by setting 80% of the minimum measured concentration for a given lipid species from all samples.

### Statistical analysis

Statistically significant differences in lipidomic profiles of healthy controls and cancer patients were evaluated using univariate methods, such as *p*-value (two-sided *T*-test or Welch test), *T*-value, and fold change (FC). Lipid species with FC (tumor/normal) greater than 1.2 or less than 0.8 (change of > 20%) and *p*-value < 0.05 were considered statistically significant. Box plots were used for the visualization of concentration changes for the most dysregulated lipid species, and statistical significance was indicated using *p*-values from the non-parametric Mann-Whitney *U* test above the box plots, with the number of significance symbols corresponding to the following ranges: *p* > 0.05 (ns, non-significant), 0.05–0.01 (*), 0.01–0.001 (**), 0.001–0.0001 (***), and < 0.0001 (****). The heat map, in combination with cluster analysis, visualizes lipid concentrations in biological samples using a color scale. The most dysregulated lipid species were visualized using a volcano plot. Visualizations were prepared in the R free software environment (ver. 4.4.1), and Cytoscape software (v. 3.8.2) was used to build a network map.

Multivariate data analysis (MDA) was performed with SIMCA 13.0.3 software (Umetrics, Sweden), including logarithmically transformed and Pareto scaled concentrations. Statistical models based on unsupervised principal component analysis (PCA) and supervised orthogonal partial least squares discriminant analysis (OPLS-DA) were performed with a seven-fold cross-validation approach. The most dysregulated lipid species were verified using the S-plot generated from OPLS-DA. The variable importance in projection (VIP) scores were evaluated for each OPLS-DA statistical model, and lipid species with VIP values higher than 1 were considered statistically relevant.

## Results and discussion

### Method optimization

This work follows our previous study focusing on the development of a derivatization method using benzoyl chloride [22], which showed significantly decreased LOD for multiple lipid classes, especially DG, MG, ST, and SPB. The aim of this work is to transfer the untargeted method using high-resolution (HR) MS to targeted quantitation using low-resolution (LR) MS due to the high potential of derivatization tags for qualitative and quantitative analysis. The application of selective MRM transitions can lead to higher analytical sensitivity, especially for DG, MG, FA, and ST, for which suitable transitions are not available without the derivatization. Although the same separation method was used, the optimization of MS parameters and the evaluation of the behavior of derivatives were performed using at least one standard from each lipid class.

In total, 30 IS representing 16 lipid subclasses were used for the method optimization. Although chemical derivatization provides adverse side reactions, fully derivatized molecules were primarily used for the detection, except for ceramides (Cer), hexosylceramides (HexCer), and SM. Since the reaction with the secondary amines is only partial for these lipid classes, derivatives targeting only the free hydroxyl groups were selected. The [M+H]^+^ and [M+NH_4_]^+^ adducts were preferentially used for the determination, except for HexCer, where the formation of [M-C_7_H_6_O_2_+H]^+^ ion was more favorable. Selected forms of derivatives and their adducts are summarized in Table S4. However, the detailed structural characterization of derivatives was thoroughly described in the previous article [22].

**Fig. 1:**
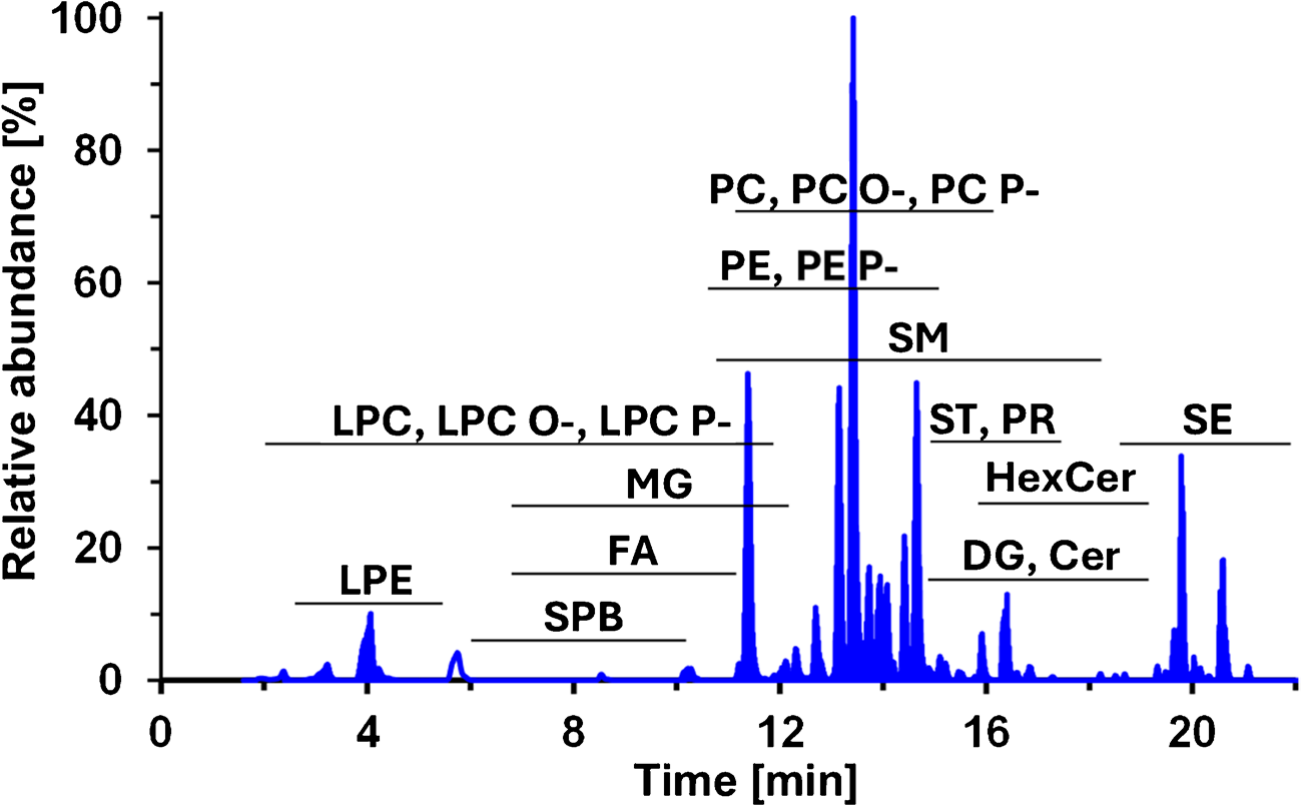
Chromatogram of human serum after derivatization with benzoyl chloride, measured using RP-UHPLC/MS/MS in the positive ion mode

The MS parameters, such as capillary voltage, source temperature, and gas pressures, were set based on the conventional lipidomic method that used the same separation method [29]. The parameters highly corresponding to the structure of analytes were optimized, such as collision energy (5–80 eV) and declustering potential (0–300 V). The values leading to the highest response were selected as optimal, except for PC and cholesterol esters, where the responses were intentionally decreased by non-optimized collision energies due to their high concentration or high ionization efficiency leading to saturation of the detector. The dwell weights (DW) were set for individual lipid classes based on concentrations of endogenous lipid species, favoring less abundant classes, such as lysophosphatidylethanolamine (LPE) or SPB. Different DW values within the same lipid class were assigned to low-abundance free sterols and their esters, in contrast to high-abundance cholesterol and cholesterol esters. The MS parameters, retention times, and MRM transition for IS were summarized in Table S4, representing settings for the individual lipid classes that allow the partial compensation of the concentration variability of the human lipidome and determination of both low- and high-abundant lipid classes together (Fig. 1).

### Identification of lipids

The pooled sample of human serum, including samples from 30 males and 30 females, was prepared as the representative matrix. The identification was performed manually using our internal lipid database. PIS, NLS, and MRM scans were used based on characteristic fragment ions. The lipid species level and molecular species level were obtained based on the type of scan, and the shorthand nomenclature for lipids was used according to Liebisch et al*.* [31]. The species level reflects the total number of carbon atoms and double bond(s), while the molecular species level provides detailed information about the composition of individual fatty acyls. Although PC and sterol esters (SE) do not undergo the derivatization reaction because they do not contain any free hydroxyl and amine groups, they can be determined together with derivatives in one analysis using traditional PIS *m*/*z* 184 for PC and *m/z* 369 for cholesterol esters. Despite the derivatization reaction, the same PIS ion *m/z* 184, as in the native form, was used for lysophosphatidylcholine (LPC) and SM; however, PIS and NLS, including derivatization tags, were used for the other lipid classes. The following scans were used for identification at the lipid species level: PIS at *m*/*z* 105 for FA, and at *m/z* 148 for LPE and PE; NLS of benzoic acid for MG, DG, SPB, Cer, ST, and prenols (PR); and NLS of the derivatized sugar unit for HexCer were used for the identification based on the lipid species level. For the fatty acyl level, NLS of fatty acids for DG and PIS of sphingoid bases for SM, Cer, and HexCer were used. Moreover, the dilution series, including five concentration levels (data are not shown), and the polynomial dependencies of retention times on the length of the fatty acyl chain (Fig. S1) and on the double bond(s) number (Fig. S2) supported the high-confidence identification. In total, 450 lipid species from 19 lipid subclasses were annotated in the pooled human serum (Table S5).

The intra-laboratory comparison of conventional and derivatization approaches using the same LC/MS platform and the use of LR- and HR-MS (QqQ vs*.* QTOF) for the same derivatization method clearly shows the benefits and limitations of the current method. The LR-MS platform confirmed the assumptions for higher sensitivity, and more identifications were observed for almost all lipid classes, resulting in more than double (450 vs. 169) identifications compared to the QTOF platform using the derivatization approach [22]. However, this comparison is partly skewed because some lipid classes, such as PC, PC P-/O-, and SE, which account for 136 species in the LR-MS method, were not investigated in the QTOF study. Compared to the HR-MS platform using the conventional approach (450 vs. 513) measured in both positive and negative ion modes [28], the higher numbers of identifications for MG, DG, HexCer, SM, ST, and SE by the current method were observed. Moreover, the derivatization method enabled the analysis of FA in the positive ion mode compared to other methods that were forced to switch to the negative ion mode. In contrast, triacylglycerols (TG) were not targeted in the current study, although they constitute a large number of identifications in conventional methods. This exclusion was because the typical MRM analysis for TG, based on the neutral loss of fatty acyls, is not specific enough to handle their structural complexity. However, the most illustrative comparison is based on the same LC/MS platform and analytical method [29]. The comparison of the same LC/LR-MS platform using the conventional approach (450 vs. 455) showed an improvement of the current method for FA, MG, DG, HexCer, SM, SPB, ST, and PR, and worse performance for LPC, LPE, PE P-/O-, phosphatidylinositols (PI), lysophosphatidylinositols (LPI), dihexosylceramides (Hex2Cer), and sulfatides (SHexCer). For other lipid classes, the numbers of identifications were comparable (Table S6).

The inter-laboratory comparison only based on the number of identifications can be inaccurate due to different LC/MS platforms, extraction methods, and the focus of the methods, but three additional RP-UHPLC/MS/MS studies focusing on complex lipidomic analysis were used for the comparison. Huynh et al*.* [32] reported 636 identified lipid species, and higher numbers were observed especially for LPC, PC P-/O-, LPE, and PE P-/O-. The other two methods [33, 34] reported 272 and 396 lipid species, and higher numbers were reported by Lerner et al*.* [34] for LPC, PC, and PC P-/O-, but the numbers of identified lipids for other lipid classes were comparable or higher in the current method. The current method showed improvements for the analysis of FA, MG, DG, HexCer, SM, SPB, SE, ST, and tocopherols compared to all inter-laboratory methods. Both intra-laboratory and inter-laboratory comparisons are summarized in Table S6.

In general, the derivatization method allows the identification of significantly higher numbers of MG, DG, SM, SPB, and ST compared to conventional lipidomic analysis. Moreover, a higher number of SE was identified, which is not caused by the derivatization reaction but by the exclusion of the MRM transition for TG eluting at the same time and focusing on other SE than cholesterol esters, such as desmosterol and sitosterol esters. However, the TG class makes up a significant part of lipids identified by conventional methods, which increases the total number of identifications. Additionally, the derivatization method enables the detection of fatty acids in the positive ion mode, including selective PIS. In contrast, the limitation of the current method is its application to lipids containing a higher number of free hydroxyl groups, such as glycosylated ceramides, and the charge-switch of PI and LPI to the positive ion mode, where they undergo in-source fragmentation. The method also showed a lower separation efficiency for *sn*−1 and *sn*−2 isomers for LPC and DG derivatives, which, unlike the conventional method, were not distinguished. A lower number of identifications was observed especially for PE P-/O-, where lower sensitivity for derivatives was already described during the method development [22].

### Validation and quantitative analysis

The method was validated before the quantitative analysis, and recommendations for bioanalytical validations were followed as closely as possible [27]. Unfortunately, it was not possible to evaluate the parameters using post-extraction spiking, such as extraction recovery and matrix effect, because derivatized IS are not available. Repeatability, instrument precision, accuracy, precision, selectivity, and carry-over were evaluated using human serum spiked with IS-Mix before protein precipitation. The validation was performed for the individual IS, and two IS were used for almost each lipid class. In total, 30 IS from 16 lipid subclasses, including deuterated and exogenous lipid species with shorter, longer, or odd carbon fatty acyls, were used (Table S1). The calibration curves were prepared based on the measurement of 16 concentration levels in triplicates, and the concentration ranges of IS were set close to physiological concentrations of lipids in human serum. Different concentrations for individual IS within the lipid class were set for SE and ST due to the broad dynamic range of these endogenous lipid classes, such as cholesterol esters (CE) vs. desmosterol esters (DE) and cholesterol vs. other ST.

**Fig. 2:**
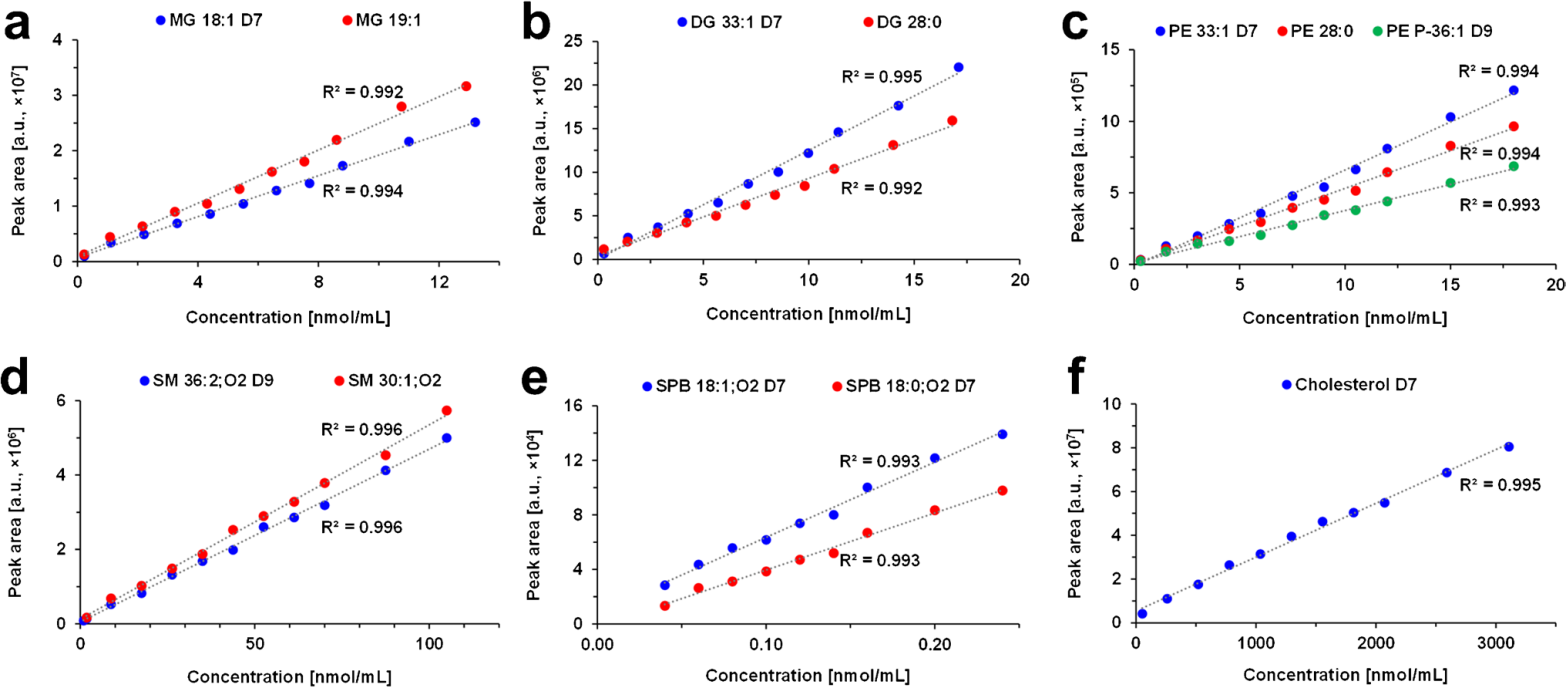
Calibration curves of internal standards after derivatization with benzoyl chloride for **a** monoacylglycerols (MG), **b** diacylglycerols (DG), **c** phosphatidylethanolamines (PE), **d** sphingomyelins (SM), **e** sphingoid bases (SPB), and **f** free sterol

The calibration range, LOD, and LOQ are reported in Table S7. Importantly, LOD and LOQ values were confirmed by direct experimental measurements rather than being calculated theoretically from calibration parameters. Calibration curves (Fig. 2 and S3) provide linear regression coefficients greater than 0.99 for all investigated analytes, except for FA, which did not pass the validation. Repeatability, accuracy, and precision were examined at low, medium, and high concentration levels, corresponding to 10, 20, and 40 μL of the IS-Mix, respectively. Accuracy and precision were evaluated in triplicate, and repeatability in six independent experiments at each concentration level. For all three parameters, the relative standard deviation (RSD) was below 10% for the most IS, and below 15% for all IS. Selectivity was determined for randomly selected samples of 4 females and 4 males by comparing the responses in blank matrix samples and matrix samples spiked with the low concentration of IS-Mix prior to extraction. The response in blank matrix samples was below 5% of the response in spiked samples for most IS. However, DG 28:0 and LPE 14:0 exceeded the 20% acceptance criterion and were thus excluded from the quantitative analysis. To evaluate the carry-over effect, solvent blanks were injected after measurements of the highest calibration level in triplicates. The response in the solvent blanks was related to the response at low concentration levels and all values were below 0.1% with the exception of SE below 1%. The instrument precision was evaluated based on medium concentration levels and all RSD values were below 10%. The results for validation parameters are summarized in Table S8.

The derivatization method improved mainly the analysis of MG, SPB, and ST, which were not detected by the conventional method using the same platform. Based on the determined LOQ values (Table S9), the sensitivity was comparable to the conventional method for most lipid classes. The exceptions were PE, PE P-, LPE, and LPC, where the derivatization reaction either decreased the sensitivity or formed two series of products [22]. However, the validation confirmed the reproducibility of the method for these lipid classes as well. Moreover, the current method enables the quantitation of DG by NLS of benzoic acid compared to neutral losses of fatty acids by the conventional method, which are not optimal for the quantitative analysis [12, 13]. The comparison of LR- and HR-MS platforms for the derivatization method showed significantly 20- to 500-fold higher sensitivity in favor of the LR platform, and the most improvements were observed for MG, PC, PE, HexCer, SM, and ST.

**Fig. 3:**
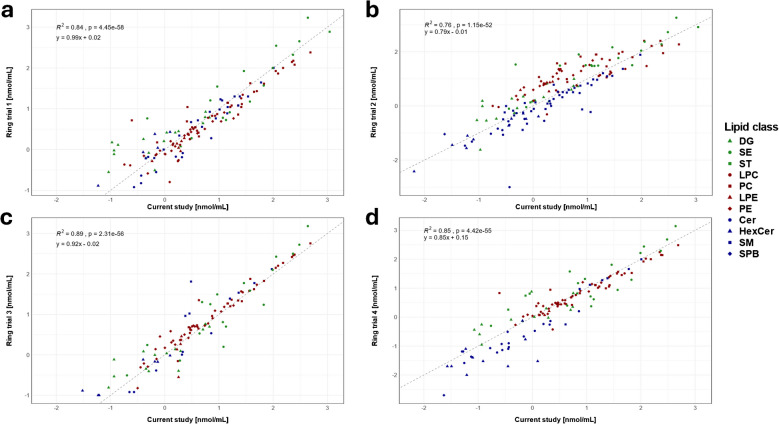
Correlation graphs for NIST SRM 1950 human plasma with the comparison of concentrations determined by the current method with those from four inter-laboratory ring trials: **a** Bowden et al*.* (ring trial 1) [35], **b** Quehenberger et al*.* (ring trial 2) [36], **c** Ghorasaini et al*.* (ring trial 3) [37], and **d** Mandal et al*.* (ring trial 4) [38]

The quantitative analysis of NIST SRM 1950 was performed, and 320 lipid species from 17 lipid subclasses were quantified (Table S10). The determined concentrations of individual lipid species were compared with the literature values of previous ring trials [35–39], which showed comparable results visualized by correlation graphs (Fig. 3). The best correlation (*R*^2^ = 0.89) was observed with Ghorasaini et al*.* [37], but high correlations were also confirmed for studies reported by Mandal et al*.* [38] and Bowden et al*.* [35] (*R*^2^ = 0.85 and *R*^2^ = 0.84, respectively). There is no significant uncorrelated lipid class; only Cer provided slightly higher and DG slightly lower concentrations compared to other studies. The lowest correlation (*R*^2^ = 0.76) was observed for the study by Quehenberger et al*.* [36], particularly for PE and PE P-/O-, where similarly poor correlations of the LipidMaps study and other ring trials were found. Overall, our results were well correlated with the ring trial data, but there are still some differences. The deviations can be caused by different platforms, extraction procedures, methods, and mainly the varying composition of IS [40]. However, there are also differences among the values reported in the literature, and accurate reference material values are needed for a reliable comparison. The detailed comparison of the concentrations of lipid species is listed in Table S10.

### Lipidomic profiling of healthy controls and cancer patients

**Fig. 4:**
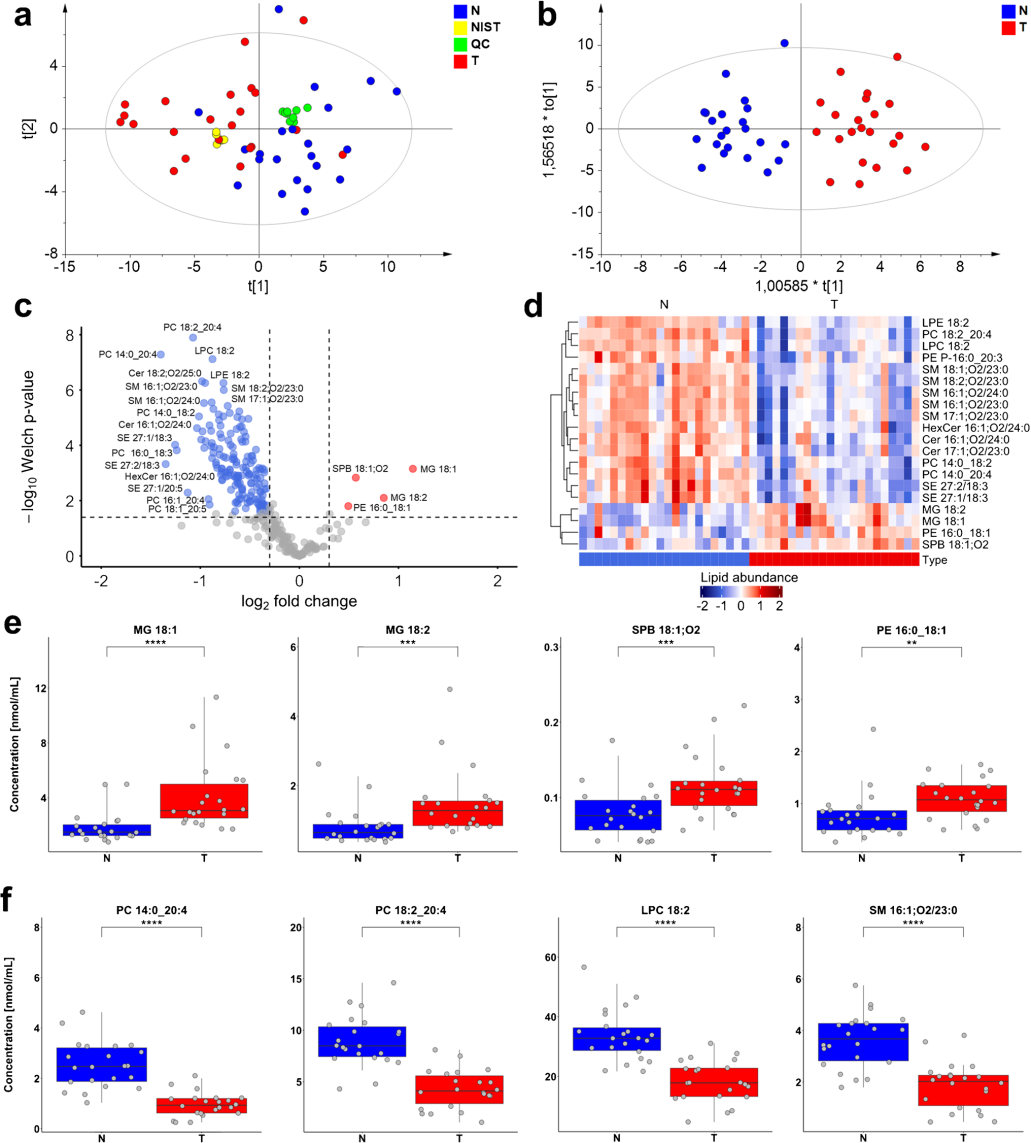
Results of the lipidomic profiling of PDAC patients (T, tumor) and healthy controls (N, normal) visualized by **a** a non-supervised PCA model, **b** supervised OPLS-DA model, **c** volcano plot with the annotation of the most upregulated (red) and downregulated (blue) lipids in PDAC, **d** heat map for selected dysregulated lipid species, **e** box plots for upregulated lipid species, and **f** box plots for selected downregulated lipids, where the number of significance symbols corresponds to the following ranges of *p*-values from the Mann-Whitney *U* test: 0.01–0.001 (**), 0.001–0.0001 (***), and <0.0001 (****)

The optimized and validated method was used to investigate differences in lipid profiles of PDAC patients and healthy controls. The inclusion criterion for healthy controls was the absence of any lifetime history of cancer. No restrictions were placed on other diseases. The samples were prospectively collected from patients with a confirmed diagnosis of PDAC, including all tumor stages (Table S3). In total, 22 healthy control samples and 22 cancer patient samples were used for the lipidomic profiling, and determined concentrations (Table S11) were evaluated using univariate (Table S12) and multivariate statistical methods. The unsupervised PCA revealed the separation between healthy controls and cancer patients, indicating differences in their lipidomic profiles (Fig. 4a). Moreover, the tight clustering of periodically injected quality control (QC) and NIST samples throughout the sequence confirmed the high quality of the data. The supervised OPLS-DA model illustrated the clear separation between both groups (Fig. 4b). Key differences were identified using S-plot generated from the OPLS-DA model and volcano plot from univariate analysis (Fig. 4c). The heat map combined with clustering analysis (Fig. 4d) and box plots (Fig. 4e, f) was used to depict the concentrations of the most dysregulated lipid species in pancreatic cancer samples. The network mapping was constructed to illustrate the dysregulation of these lipids within their metabolic pathways (Fig. S4).

Statistical analysis highlighted MG as the most upregulated lipid species, particularly MG 18:1 and MG 18:2. Although MG 16:0 and MG 16:1 showed relevant fold changes, the statistical significance was lower (Fig. S5). Other statistically significant upregulated lipids included sphingosine (SPB 18:1;O2) and PE 16:0_18:1, which is consistent with our previous results based on the lipid class separation [8]. Moreover, DG presented substantial fold changes (Fig. S5), aligning with previous reports [7], but their statistical relevance was less pronounced.

A significantly higher number of downregulated lipid species, including phospholipids, sphingolipids, and sterol esters, was identified, showing several similarities in their patterns. For phospholipids (PC, PC P-/O-, and PE P-) and lysophospholipids (LPC and LPE), dysregulated lipid species predominantly contained fatty acyl compositions of 18:2 and 20:4, either in combination with each other or with other acyl chains (Fig. S6). Moreover, all PE P- were downregulated compared to PE, which were statistically non-significant or upregulated. Statistically relevant SE included mainly fatty acyls containing 18 or 20 carbon atoms, but compositions of 18:2, 18:3, and 20:4 were the most statistically significant (Fig. S7). Based on the sterol skeleton, cholesterol and desmosterol esters showed highly statistically significant changes, while sitosterol esters showed less pronounced or non-significant changes (Fig. S4). ST showed low statistical significance, except for downregulated desmosterol, which was statistically relevant (Table S12).

**Fig. 5:**
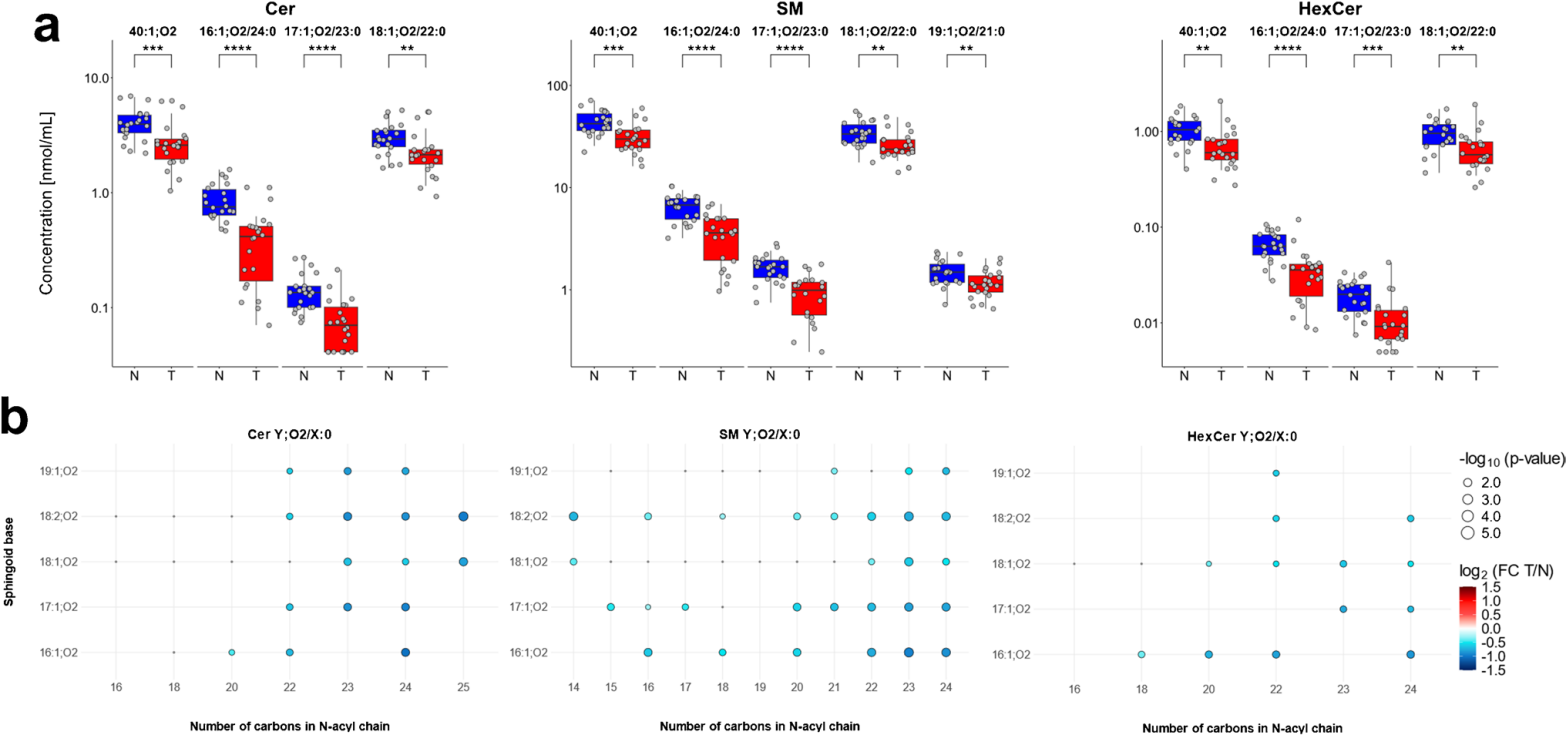
Concentration differences and statistical relevance of selected ceramides (Cer), sphingomyelins (SM), and hexosylceramides (HexCer) between healthy controls and PDAC patients: **a** comparison of the lipid species level (sum of isomers, e.g., Cer 40:1;O2) vs. the fatty acyl level (individual isomers, e.g., Cer 16:1;O2/24:0) and **b** the effect of *N*-acyl chain composition, where Y represents the sphingoid base and X denotes the number of carbons in the *N*-acyl chain

The key finding emerged in sphingolipids, especially for sphingolipids with very long *N*-acyl chains, which is demonstrated on sphingolipids with the summary composition 40:1 (Fig. 5a). This trend was also examined for other sphingolipids, such as 39:1, 41:1, 41:2, 42:1, and 42:2 (Fig. S8). Resolving the *N*-acyl chain composition plays an important role, as not all isomers show statistically significant differences in concentrations between healthy controls and cancer patients. The lipid class separation approach provides only information about total carbon number and double bond(s), without information on the structure of individual isomers, and the statistical relevance often follows the most abundant isomer. For example, the sum of isomers for SM 42:2;O2 is statistically non-significant, as well as individual isomers SM 18:1;O2/24:1 and SM 19:1;O2/23:1, but SM 18:2;O2/24:0 is statistically significant. The same pattern is observed for most sphingolipids (Fig. S9). However, the most statistically relevant sphingolipids were isomers with saturated very long *N*-acyl chains (Fig. 5b and S8), especially *N*-acyl chains with more than 20 carbons. These findings suggest alterations in ceramide metabolism in PDAC patients, characterized by overall downregulation of sphingolipids across the metabolic pathway and concurrent upregulation of sphingosine. Concentration changes of sphingolipids with saturated very long *N*-acyl chains can result from altered activity of ceramide synthase 2, which predominantly synthesizes ceramides with C22–C26 acyl chains. Since sphingolipids are involved in both apoptosis and cell signaling, these variations may contribute to cancer cell survival and uncontrolled proliferation [41].

Although the current study includes a relatively small number of samples, the results correlate with our previously reported dysregulations using lipid class separation approaches [7, 8] involving large cohorts and are consistent with the conventional method using lipid species separation approach [29]. Compared with lipid class separation approaches, the current methodology provides detailed information on the fatty acyl composition, which helps to inform the design of further experiments to investigate the biological mechanism. Furthermore, the current method provides new insights into MG, SPB, and ST, which showed statistically significant dysregulations and highlight a high potential in cancer biomarker research.

## Conclusions

We present a targeted RP-UHPLC/MS/MS method that enables the quantitation of challenging lipid classes by employing benzoyl chloride derivatization to create selective and sensitive MRM transitions, especially for species like MG, DG, SPB, and ST. The optimized method shows an improvement in the analysis of these lipid classes and a comparable sensitivity for other lipid classes compared to the conventional RP-UHPLC/MS/MS method. The establishment of highly confident identification criteria combining several parameters, such as MRM transition, retention dependencies, dilution series, and derivatization tags for 450 lipid species, and the validation of the quantitative workflow against NIST SRM 1950 demonstrate a robust platform for lipidomic analysis. Finally, the method is applied to a clinical cohort of pancreatic cancer patients and healthy controls to investigate metabolic alterations. The lipidomic profiles show differences primarily in sphingolipids and phospholipids, which are consistent with previous reports. In addition, our approach reveals new findings, including a significant upregulation of MG and SPB. The specific downregulation of sphingolipids that contain very long *N*-acyl chains is also highlighted, a structural detail often missed by methods using a lipid class separation approach. These results underscore the importance of the combination of the derivatization and RP-UHPLC/MS/MS methods, which lead to highly sensitive and selective analysis. The presented methodology has great potential for larger clinical studies aimed at the discovery of novel cancer biomarkers and a deeper understanding of metabolic shifts in cancer.

## Supplementary Information

Below is the link to the electronic supplementary material.

Supplementary Material 1 (PDF 1.24 MB)

Supplementary Material 1 (XLSX 773 KB)

## Data Availability

The authors declare that the data supporting the findings of this study are available within the paper and its Supplementary Information files. Raw data files are available from the corresponding author upon reasonable request.

## Abbreviations

ACN: Acetonitrile
BMI: Body mass index
Bz-Cl: Benzoyl chloride
CE: Cholesterol ester
Cer: Ceramide
DE: Desmosterol ester
DG: Diacylglycerol
DP: Declustering potential
DW: Dwell weights
FA: Fatty acid
FC: Fold change
Hex2Cer: Dihexosylceramide
HexCer: Hexosylceramide
HR: High-resolution
IPA: 2-Propanol
IS: Internal standard
IS-Mix: Internal standard mixture
LOQ: Limit of quantitation
LPC: Lysophosphatidylcholine
LPE: Lysophosphatidylethanolamine
LPI: Lysophosphatidylinositol
LR: Low-resolution
MDA: Multivariate data analysis
MeOH: Methanol
MG: Monoacylglycerol
MRM: Multiple reaction monitoring
MS: Mass spectrometry
N: Normal (healthy control)
NLS: Neutral loss scans
ns: Non-significant
-O: Alkyl bond
OPLS-DA: Orthogonal partial least squares discriminant analysis
-P: Plasmalogen
PCA: Principal component analysis
PC: Phosphatidylcholine
PDAC: Pancreatic ductal adenocarcinoma
PE: Phosphatidylethanolamine
PI: Phosphatidylinositol
PIS: Precursor ion scans
PR: Prenol
Pyr: Pyridine
QC: Quality control
QqQ: Triple quadrupole
QTOF: Quadrupole – time-of-flight
QTRAP: Quadrupole-linear ion trap
RP-UHPLC/MS/MS: Reversed-phase ultrahigh-performance liquid chromatography tandem mass spectrometry
RSD: Relative standard deviation
SE: Sterol ester
SHexCer: Sulfatide
SM: Sphingomyelin
SPB: Sphingoid base
SRM: Standard Reference Material
ST: Free sterol
T: Tumor (PDAC patient)
TG: Triacylglycerol
UPHLC/MS: Ultrahigh-performance liquid chromatography mass spectrometry
VIP: Variable importance in projection
