# Supplementary material for: Benzoyl chloride derivatization improves selectivity and sensitivity of lipidomic quantitation in human serum of pancreatic cancer patients using RP-UHPLC/MS/MS
Source: Anal Bioanal Chem. 2025 Oct 6;418(2):733–45. doi: 10.1007/s00216-025-06151-0 (PMC12783317; doi:10.1007/s00216-025-06151-0)
Supplement: Supplementary file 1 — Supplementary Material 1 (PDF 1.24 MB) [file 216_2025_6151_MOESM1_ESM.pdf]

Supplementary material

Benzoyl Chloride Derivatization Improves Selectivity and Sensitivity of Lipidomic Quantitation in Human Serum of Pancreatic Cancer Patients Using RP-UHPLC/MS/MS

Ondřej Peterka[1], Zuzana Lásko[1], Robert Jirásko[1], Petra Peroutková[1], Anna Taylor[1], Beatrice Mohelníková-Duchoňová[2], Irena Kozubíková[2], Martin Loveček[3], Bohuslav Melichar[2], and Michal Holčapek[1,*]

[1]University of Pardubice, Faculty of Chemical Technology, Department of Analytical Chemistry, Studentská 573, 53210 Pardubice, Czech Republic

[2]Palacký University Olomouc and University Hospital, Faculty of Medicine and Dentistry, Department of Oncology, Zdravotníků 248, 775 20 Olomouc, Czech Republic

[3]Palacký University Olomouc and University Hospital, Faculty of Medicine and Dentistry, Department of Surgery, Hněvotínská 3, 779 00 Olomouc, Czech Republic

*Corresponding author: Michal Holčapek, Tel.: +420466037087; Fax: +420466037068; Email: Michal.Holcapek@upce.cz

Table of content

Fig. S1: Graphical visualization of dependence of retention times on the carbon number, where X represents the carbon number. The number of double bond(s) for each lipid is written after the colon. 4

Fig. S2: Graphical visualization of dependence of retention times on number of double bond(s), where Y represents the number of double bond(s). The total carbon number for each lipid is written before the colon. 6

Fig. S3: Calibration curves of derivatized internal standards in spiked human serum. Data present the mean value of three independent experiments. 8

Fig. S4: Network mapping of lipid species for: **a)** phospholipids and glycerolipids, **b)** sphingolipids, and **c)** sterols, where the size of each circle reflects *p*-values for individual lipids, and red/blue color saturation represents fold change (T/N). 9

Fig. S5: Box plots of upregulated glycerolipids showing differences in concentrations between healthy controls (N, blue) and PDAC patients (T, red). 10

Fig. S6: Box plots of the most downregulated phospholipids showing differences in concentrations between healthy controls (N, blue) and PDAC patients (T, red). 11

Fig. S7: Box plots of the most downregulated sterol esters showing differences in concentrations between healthy controls (N, blue) and PDAC patients (T, red). 12

Fig. S8: Statistical significance for: **a)** ceramides, **b)** sphingomyelins, and **c)** hexosylceramides based on fatty acyl compositions. 13

Fig. S9: Box plots visualizing the effect of fatty acyl composition on the statistical significance of lipid species for: **a)** ceramides, **b)** hexosylceramides, and **c)** sphingomyelins. 14

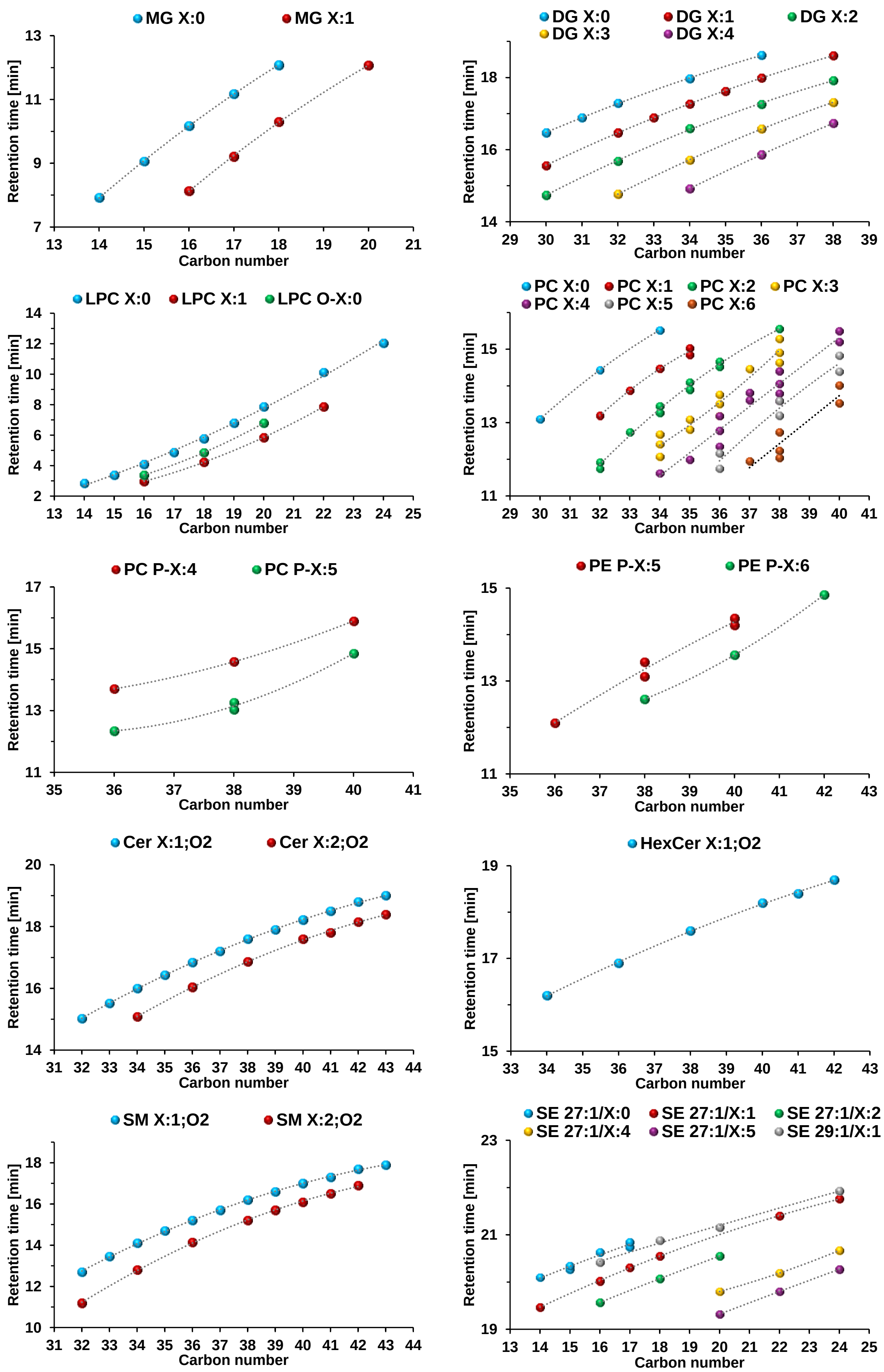

MG X:0
MG X:1
Retention time [min]
13
11
9
7
13 14 15 16 17 18 19 20 21
Carbon number
DG X:0
DG X:1
DG X:2
DG X:3
DG X:4
18
16
14
29 30 31 32 33 34 35 36 37 38 39
LPC X:0
LPC X:1
LPC O-X:0
14
12
10
8
6
4
2
13 14 15 16 17 18 19 20 21 22 23 24 25
PC X:0
PC X:1
PC X:2
PC X:3
PC X:4
PC X:5
PC X:6
15
13
11
29 30 31 32 33 34 35 36 37 38 39 40 41
PC P-X:4
PC P-X:5
17
15
13
11
35 36 37 38 39 40 41
PE P-X:5
PE P-X:6
15
13
11
35 36 37 38 39 40 41 42 43
Cer X:1;O2
Cer X:2;O2
20
18
16
14
31 32 33 34 35 36 37 38 39 40 41 42 43 44
HexCer X:1;O2
19
17
15
33 34 35 36 37 38 39 40 41 42 43
SM X:1;O2
SM X:2;O2
18
16
14
12
10
31 32 33 34 35 36 37 38 39 40 41 42 43 44
SE 27:1/X:0
SE 27:1/X:1
SE 27:1/X:2
SE 27:1/X:4
SE 27:1/X:5
SE 29:1/X:1
23
21
19
13 14 15 16 17 18 19 20 21 22 23 24 25

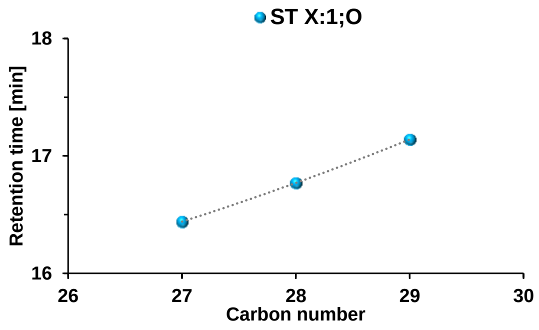

Figure S1: Graphical visualization of dependence of retention times on the carbon number, where X represents the carbon number. The number of double bond(s) for each lipid is written after the colon.

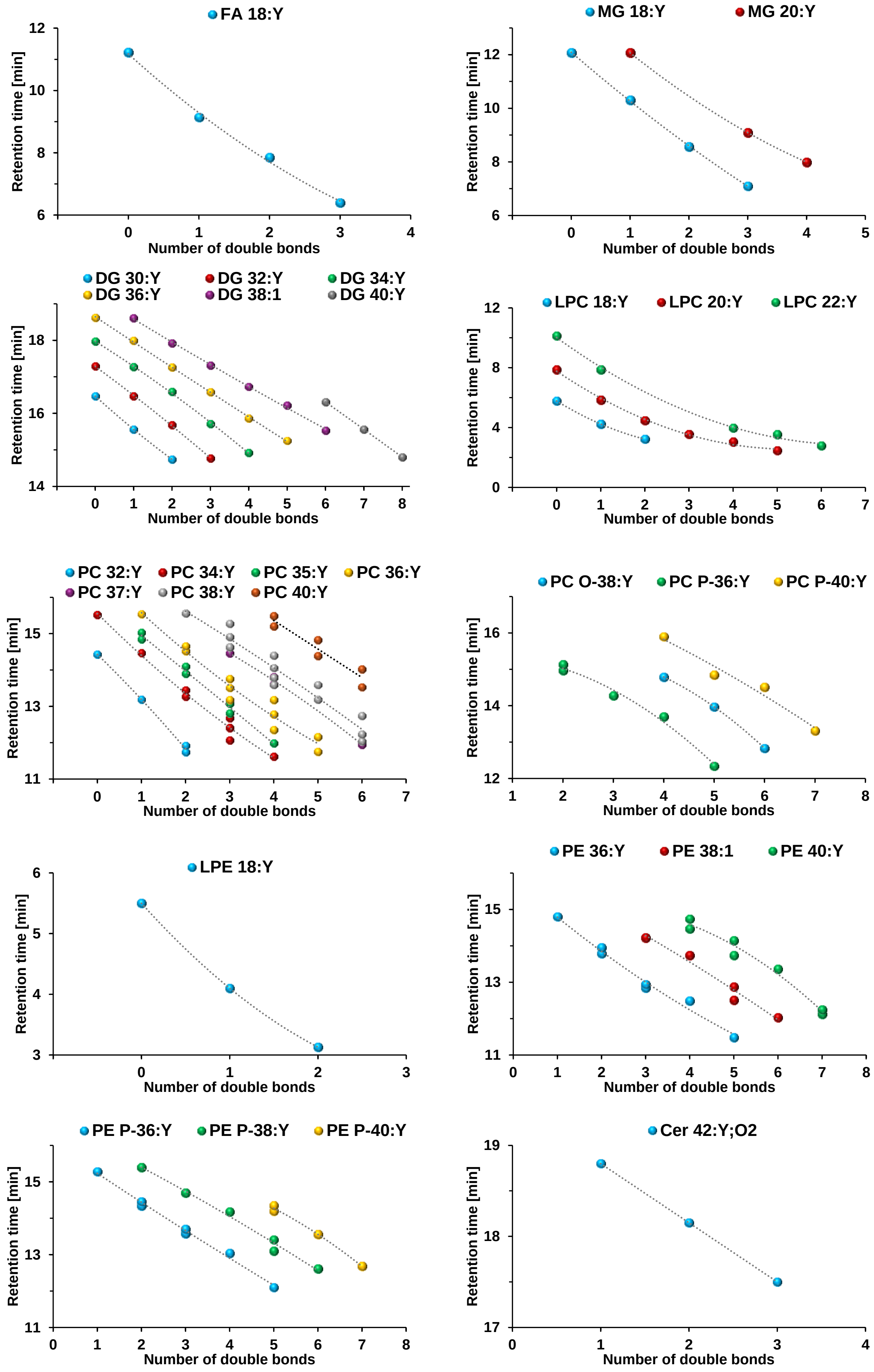
FA 18:Y
MG 18:Y
MG 20:Y
DG 30:Y
DG 32:Y
DG 34:Y
DG 36:Y
DG 38:1
DG 40:Y
LPC 18:Y
LPC 20:Y
LPC 22:Y
PC 32:Y
PC 34:Y
PC 35:Y
PC 36:Y
PC 37:Y
PC 38:Y
PC 40:Y
PC O-38:Y
PC P-36:Y
PC P-40:Y
LPE 18:Y
PE 36:Y
PE 38:1
PE 40:Y
PE P-36:Y
PE P-38:Y
PE P-40:Y
Cer 42:Y;O2
Retention time [min]
Number of double bonds

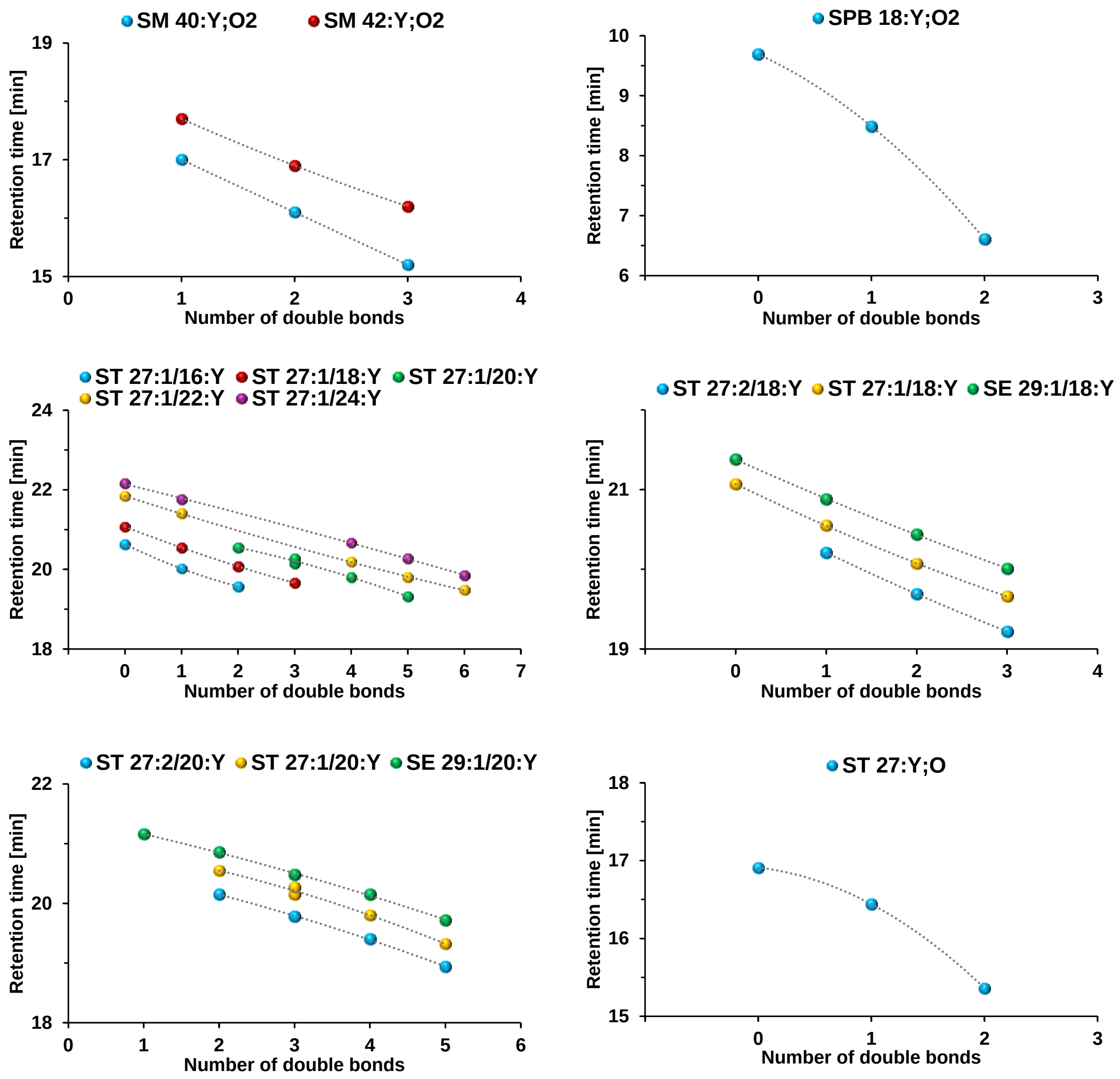

Figure S2: Graphical visualization of dependence of retention times on number of double bond(s), where Y represents the number of double bond(s). The total carbon number for each lipid is written before the colon.

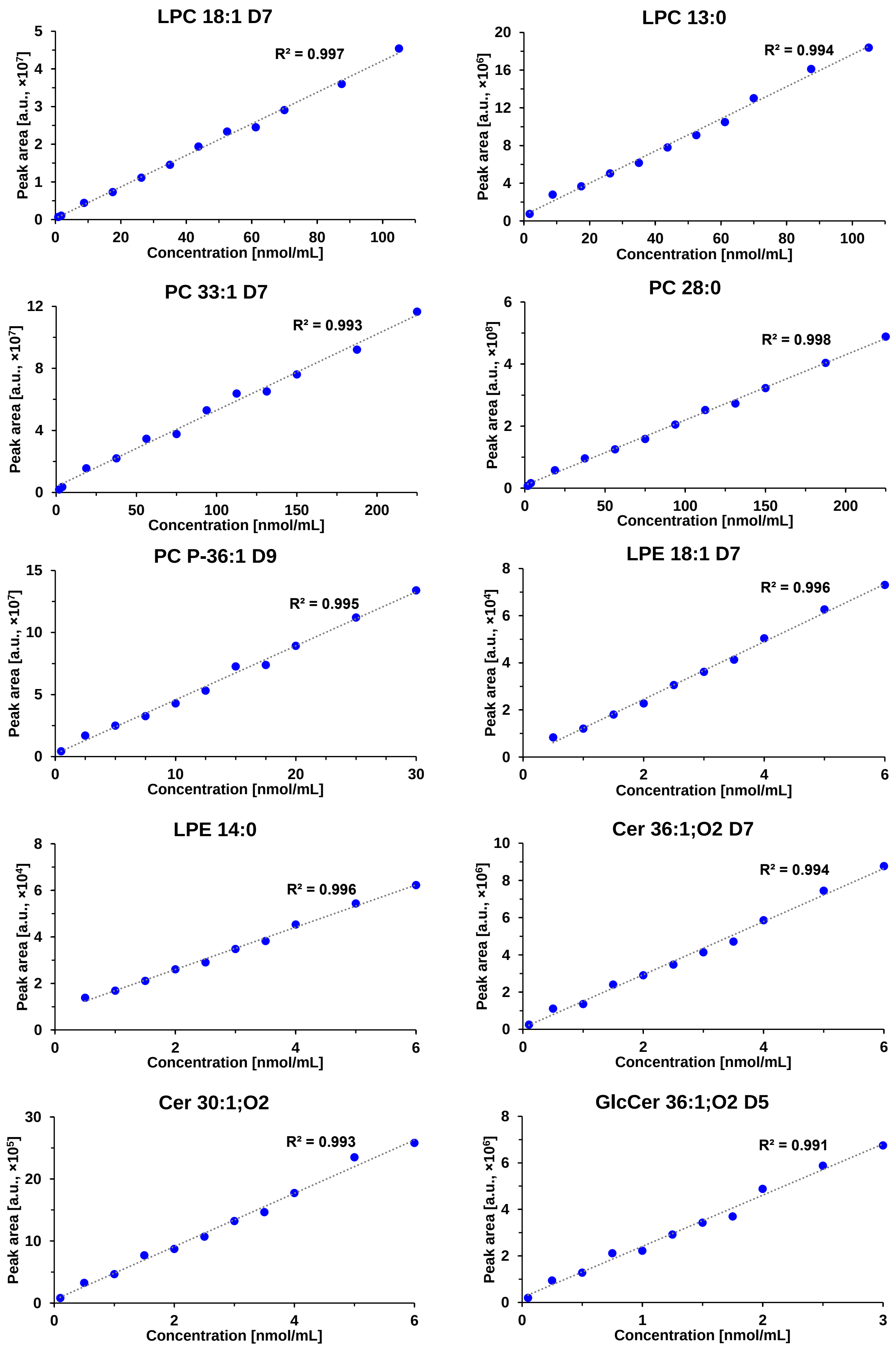

LPC 18:1 D7
R² = 0.997
Peak area [a.u., ×10⁷]
Concentration [nmol/mL]
LPC 13:0
R² = 0.994
Peak area [a.u., ×10⁶]
Concentration [nmol/mL]
PC 33:1 D7
R² = 0.993
Peak area [a.u., ×10⁷]
Concentration [nmol/mL]
PC 28:0
R² = 0.998
Peak area [a.u., ×10⁸]
Concentration [nmol/mL]
PC P-36:1 D9
R² = 0.995
Peak area [a.u., ×10⁷]
Concentration [nmol/mL]
LPE 18:1 D7
R² = 0.996
Peak area [a.u., ×10⁴]
Concentration [nmol/mL]
LPE 14:0
R² = 0.996
Peak area [a.u., ×10⁴]
Concentration [nmol/mL]
Cer 36:1;O2 D7
R² = 0.994
Peak area [a.u., ×10⁶]
Concentration [nmol/mL]
Cer 30:1;O2
R² = 0.993
Peak area [a.u., ×10⁵]
Concentration [nmol/mL]
GlcCer 36:1;O2 D5
R² = 0.991
Peak area [a.u., ×10⁶]
Concentration [nmol/mL]

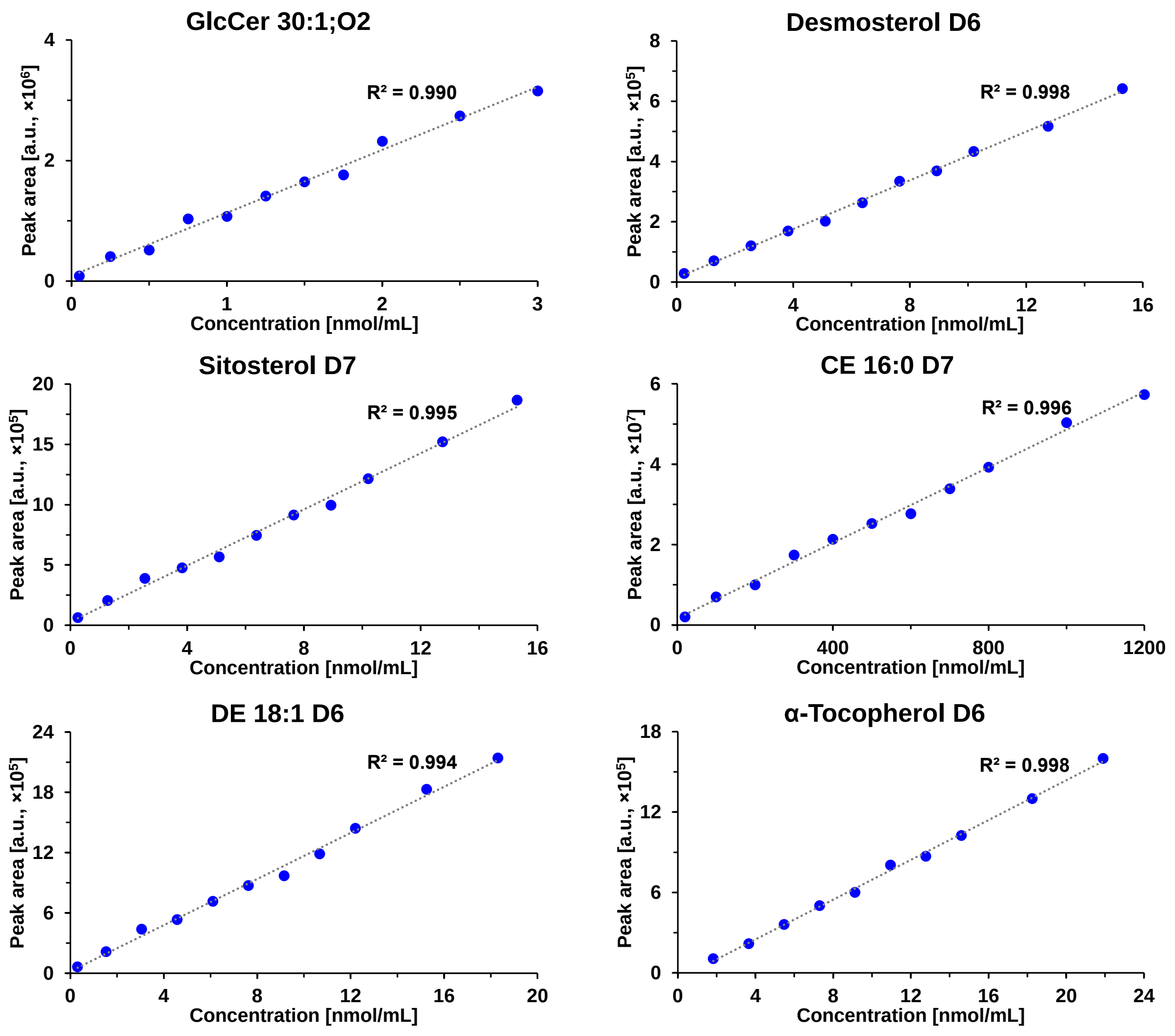

Figure S3: Calibration curves of derivatized internal standards in spiked human serum. The data represent the mean values of three independent experiments.

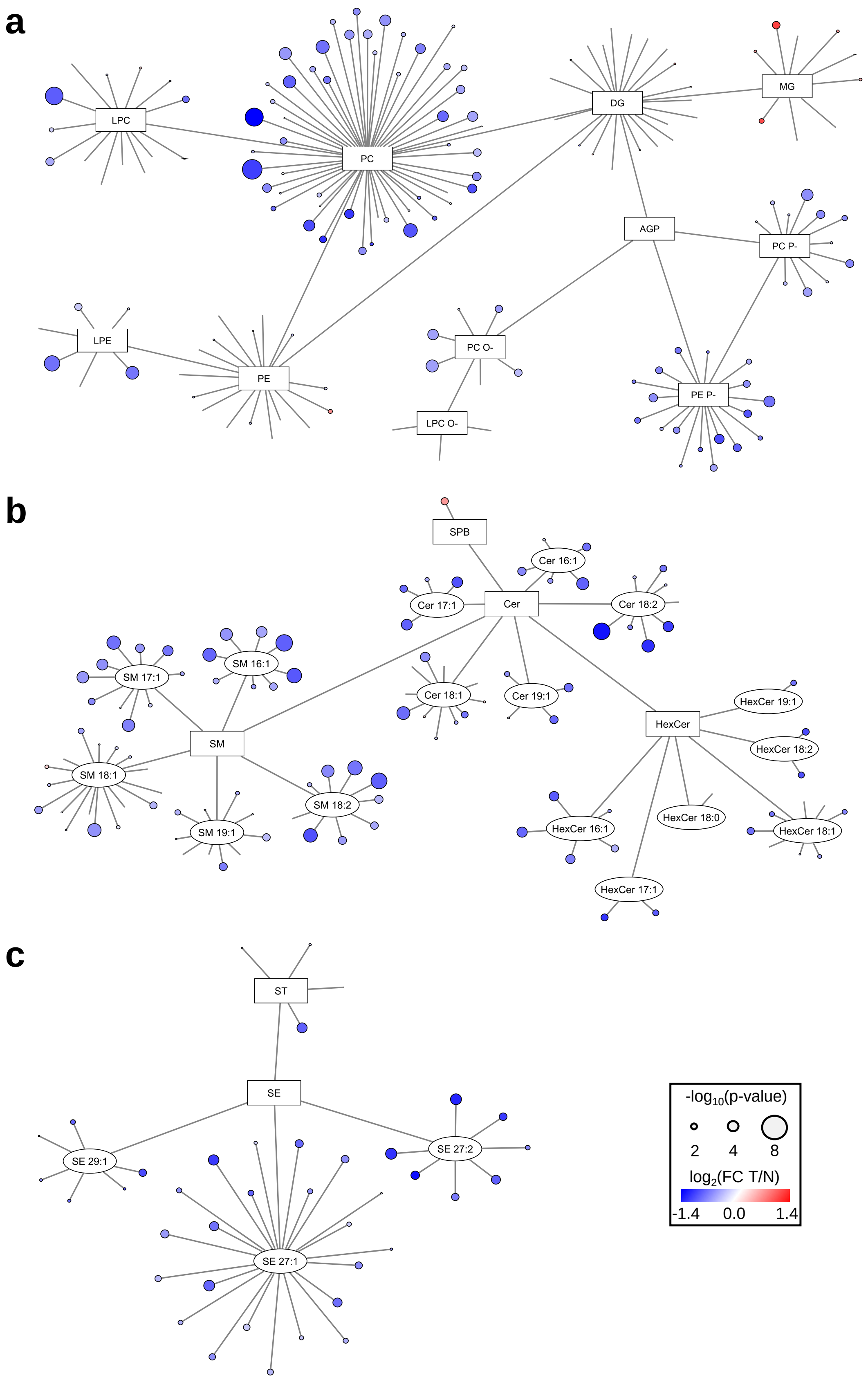

Figure S4: Network mapping of lipid species for: **a)** phospholipids and glycerolipids, **b)** sphingolipids, and **c)** sterols, where the size of each circle reflects the *p*-values for individual lipids, and red/blue color saturation represents the fold change (T/N).

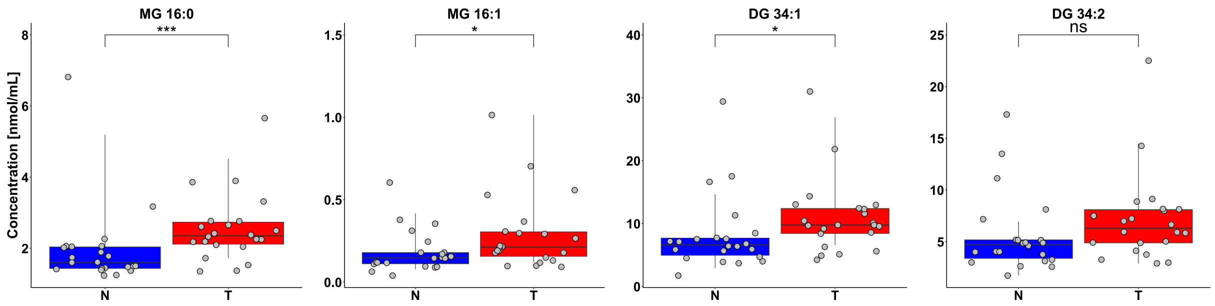

Figure S5: Box plots of upregulated glycerolipids showing differences in concentrations between healthy controls (N, blue) and PDAC patients (T, red). The number of significance symbols corresponds to *p*-value ranges from the Mann–Whitney U test, $p > 0.05$ (ns = non-significant), 0.05–0.01 (*), 0.01–0.001 (**), 0.001–0.0001 (***), and <0.0001 (****).

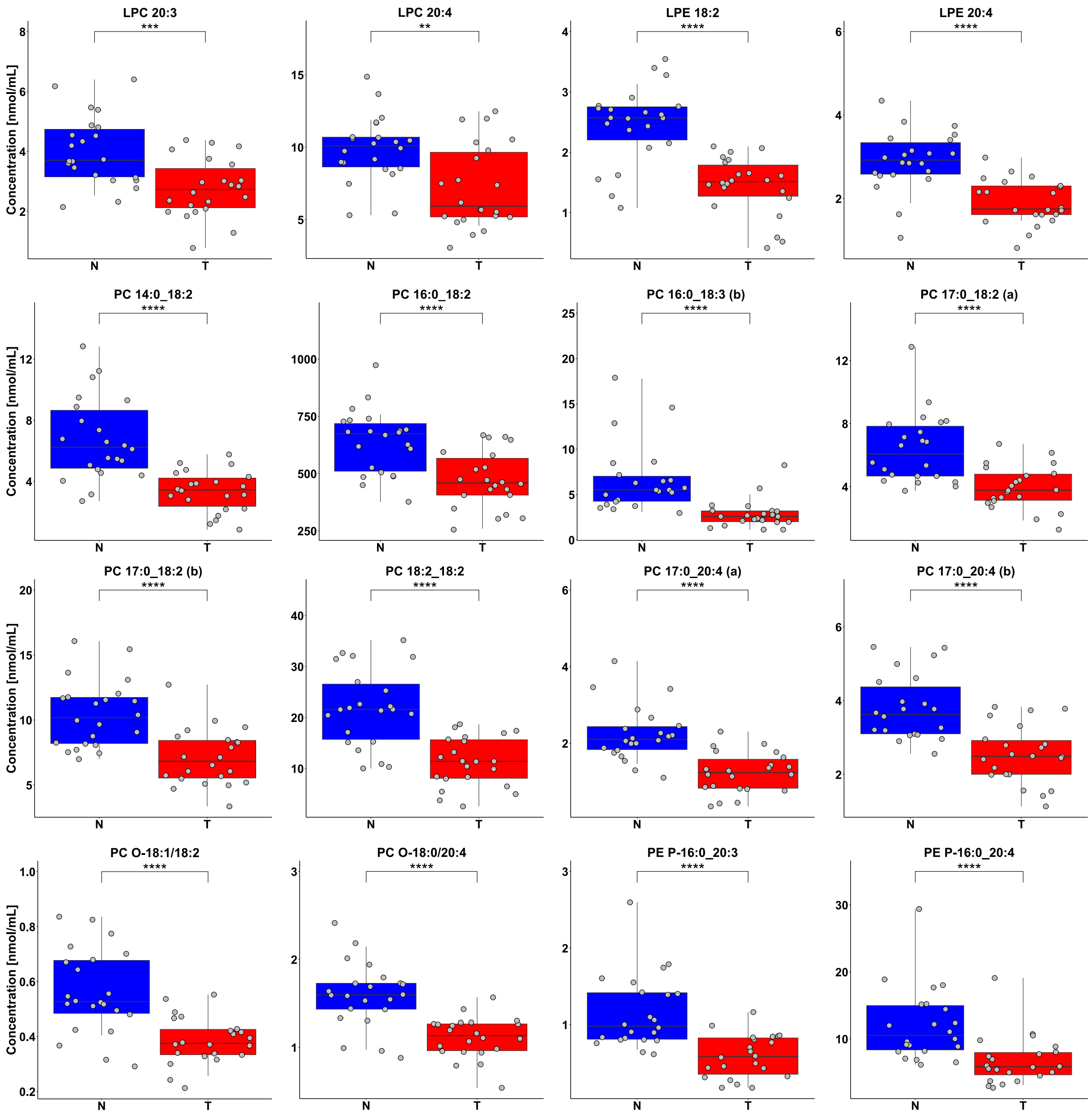

Figure S6: Box plots of the most downregulated phospholipids showing differences in concentrations between healthy controls (N, blue) and PDAC patients (T, red). The number of significance symbols corresponds to *p*-value ranges from the Mann–Whitney U test: 0.01–0.001 (**), 0.001–0.0001 (***), and <0.0001 (****).

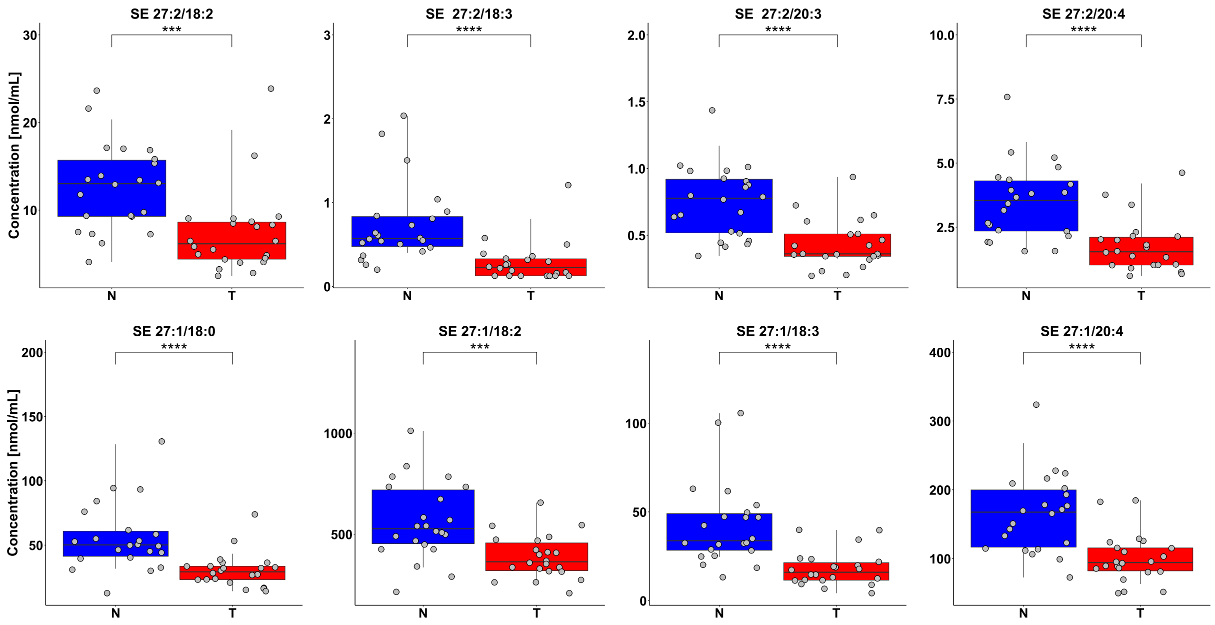

Figure S7: Box plots of the most downregulated sterol esters showing differences in concentrations between healthy controls (N, blue) and PDAC patients (T, red). The number of significance symbols corresponds to *p*-value ranges from the Mann–Whitney U test, 0.001–0.0001 (***) and <0.0001 (****).

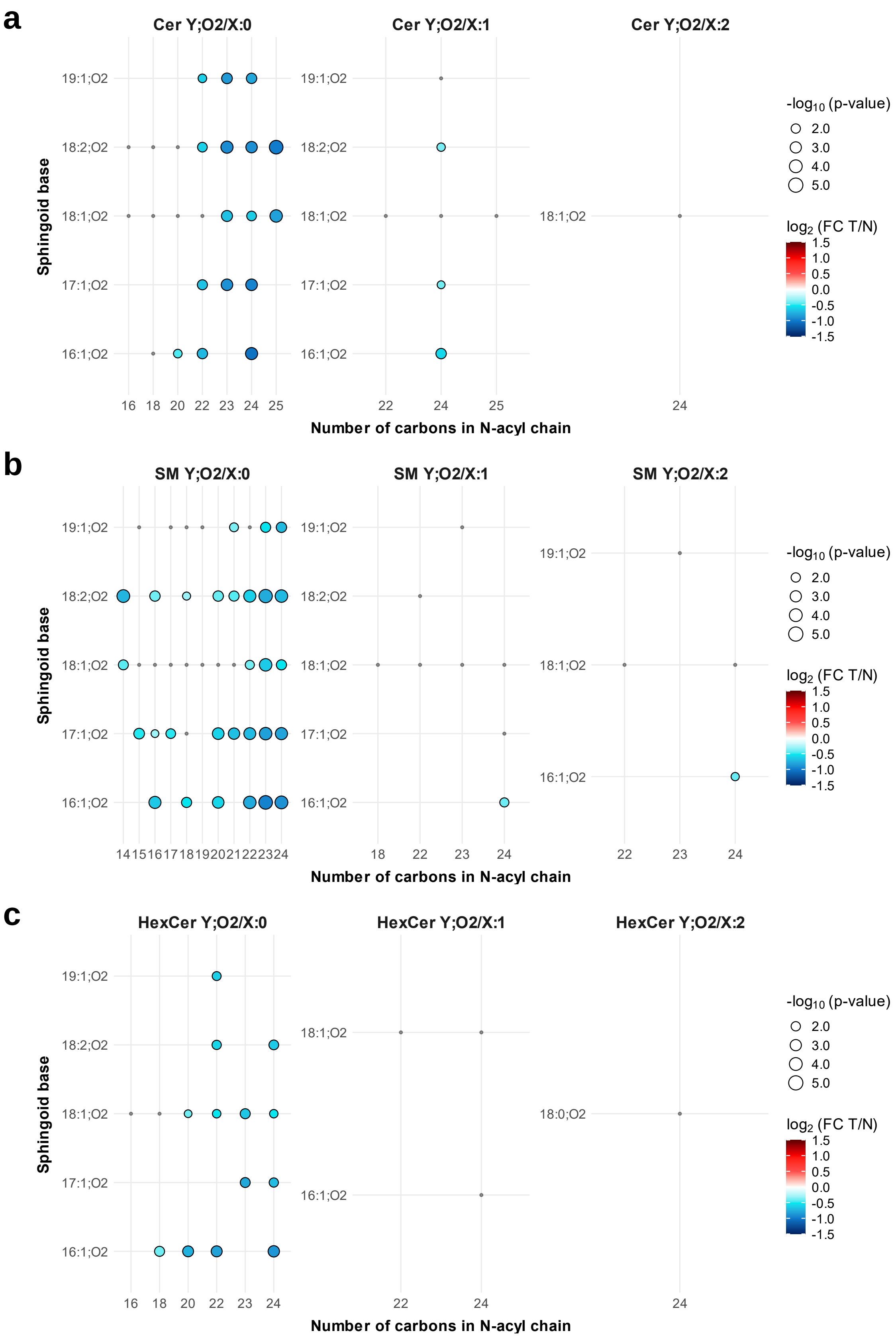

Figure S8: Statistical significance for: **a)** ceramides, **b)** sphingomyelins, and **c)** hexosylceramides based on fatty acyl compositions, where Y represents the sphingoid base and X denotes the number of carbons in the *N*-acyl chain.

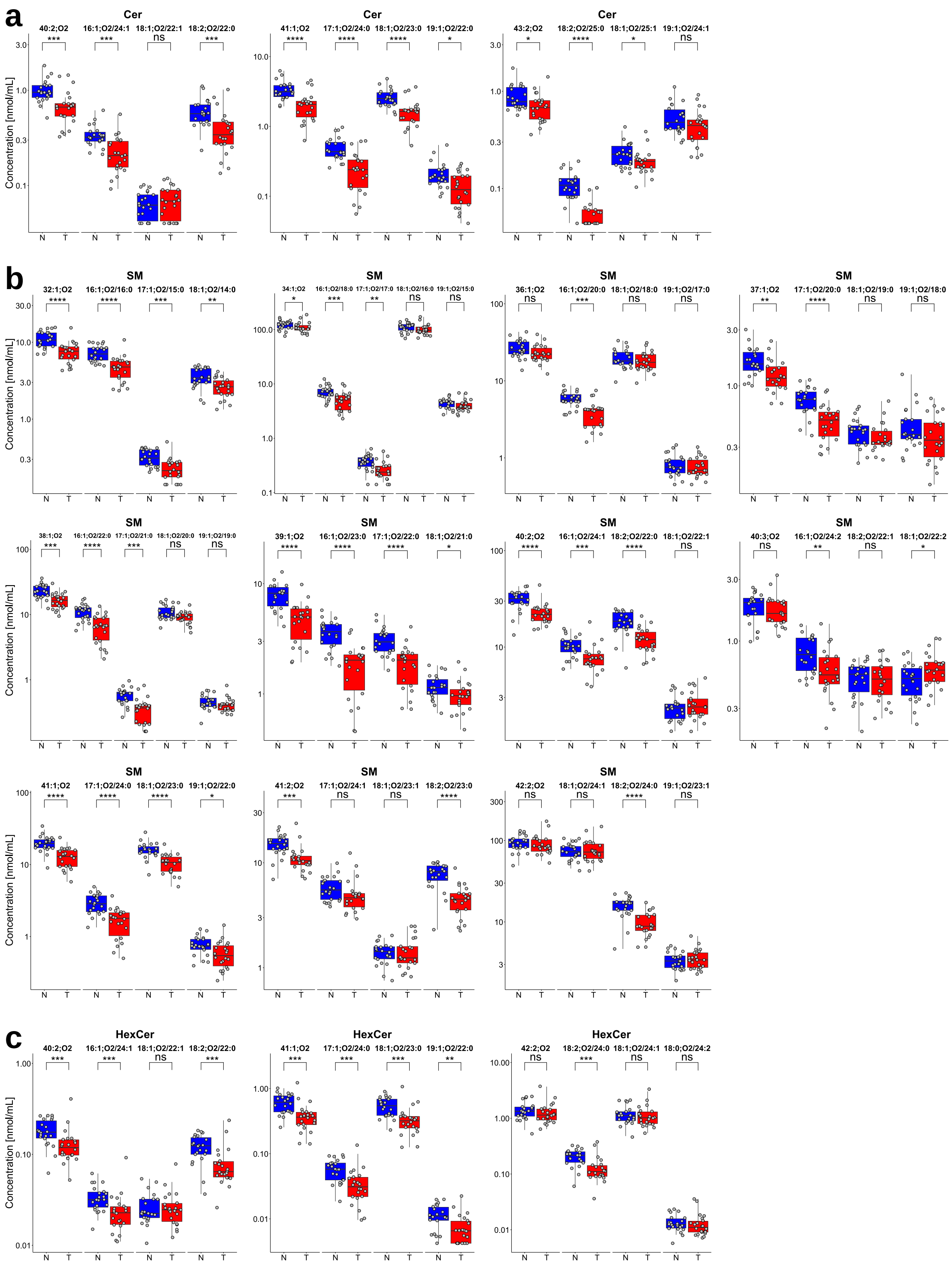

Figure S9: Box plots visualizing the effect of fatty acyl composition on the statistical significance of lipid species for: **a)** ceramides, **b)** sphingomyelins, and **c)** hexosylceramides.
